# Editorial of Special Issue “Hyaluronic Acid in Human Medicine”

**DOI:** 10.3390/biom12101495

**Published:** 2022-10-17

**Authors:** Jakub Suchánek

**Affiliations:** 1 Department of Dentistry, Faculty of Medicine in Hradec Kralove, Charles University, Simkova 870, 500 03 Hradec Kralove, Czech Republic; suchanekj@lfhk.cuni.cz; 2 Faculty of Medicine in Hradec Kralove, University Hospital Hradec Kralove, 500 05 Hradec Kralove, Czech Republic

Hyaluronic acid (HA) is an acidic, non-sulfated glycosaminoglycan that is intensively studied as a biodegradable and biocompatible material for scaffolding, regenerative medicine, and clinical applications. As the ubiquitous component of the extracellular matrix, HA is widely distributed in the human body and can be found in the umbilical cord, synovial fluid, dental pulp, vitreum, or epithelial and connective tissues. The main functions of HA are hydration, space-filling capacity, lubrication, and forming of the framework through which cells migrate. It also contributes to fetal healing of wounds, i.e., rapid healing without a scar, and tissue elasticity. However, there are additional properties of HA that are varied, based on the length of its molecular chain. In the human body, HA is synthesized and dominantly presented in high molecular weight; thus, long-chain molecules of HA are a part of the natural environment of cells. During the degradation of HA, which is accelerated under pathological conditions, its long molecules are cleaved into smaller fragments of low molecular weight. Bioactive functions in the inflammatory reaction, angiogenesis, or its role in cancer progression and reactive oxygen species scavenging vary for different fractions of HA.

The main aim of this *Biomolecules* Special Issue Hyaluronic Acid in Human Medicine was to gather the knowledge about studied potential applications of hyaluronic acid worldwide. We collected 11 articles which from 7 are original papers, 3 are reviews and 1 is a case report. The Special Issue Hyaluronic Acid in Human Medicine articles are based on preclinical and clinical research and studies. The articles include many different branches of medicine, e.g., cell banking, skin healing, vocal cord augmentation, healing of osteoarthritis, ophthalmology, lung immunity, surgery, and dentistry. Therefore, the *Biomolecules* Special Issue Hyaluronic Acid in Human Medicine represents unique outline of possible variety usage of hyaluronic acid within the latter-day and future medicine.

I would like to give a short inside view about the articles and their topics.

The article “Innovative Approach in the Cryogenic Freezing Medium for Mesenchymal Stem Cells” by Pilbauerova and co-workers [1] represents a unique preclinical study on the possible usage of hyaluronic acid during cell cryopreservation. In this study, the authors were able to reduce the concentration of toxic DMSO from widely used 10% to 2% and obtain even more viable and higher quality stem cells after defrosting. This procedure can highly affect widely use cryopreservation technique used not only during preclinical experiments, but even more during any other already established protocols in clinical medicine.

Articles “The Degradation of Hyaluronan in the Skin” [2] and “Nonwoven Textiles from Hyaluronan for Wound Healing Applications” [3] by two research groups which are connected under the Contipro a.s. company gives a comprehensive picture as to how hyaluronic acid can influence the healthy or damaged skin. The topics of these two articles were further extended by the other Contipro a.s. research group, who focused on studying the effect of hyaluronic acid conjugated with all-trans retinoic acid in micelles form on skin cells’ retinoid gene expression. In this article “Retinoic Acid Grafted to Hyaluronic Acid Activates Retinoid Gene Expression and Removes Cholesterol from Cellular Membranes” [4], the authors suggest that this substance can be used for treatment of chronic skin diseases such as psoriasis and acne. Unlike the simple all-trans retinoic acid, the all-trans retinoic acid conjugated with hyaluronic acid activated the expression of cholesterol metabolism genes.

Yi-Chieh Lee and co-workers focused on the need of secondary application of hyaluronic acid into the vocal cord in case of unilateral vocal fold paralysis. The results on the clinical trial including 94 patients found that in case of application of high-concentrated hyaluronic acid, the necessitate of a second application within 12 months was lowered from 14.1%, patients with low concentrated hyaluronic acid, to 4.3%, patients with high-concentrated hyaluronic acid. The results are published in the article “Long-Lasting Effect after Single Hyaluronate Injection for Unilateral Vocal Fold Paralysis: Does Concentration Matter?” [5].

In the article “Assessment of the Response Profile to Hyaluronic Acid Plus Sorbitol Injection in Patients with Knee Osteoarthritis: Post-Hoc Analysis of a 6-Month Randomized Controlled Trial” [6] Bruyere and co-workers have shown that hyaluronic acid plays a key role in management of osteoartrhritis. In their post hoc analysis of data from a 24 week (168 days) prospective, randomized, phase III b, double-blind, controlled trial, they proved that the hyaluronic acid is beneficial for the patient, but not as the first option. According to their results, the hyaluronic acid joint injection should be used after the symptomatic slow acting drugs in, and topical and oral NSAIDs.

As the hyaluronic acid serves as the lubricant, it is often added to eye drops to help fight against so-called ‘dry eye syndrome’, which relates to more diseases. In the original article “Insight into the Lubrication and Adhesion Properties of Hyaluronan for Ocular Drug Delivery” [7], authors present tribological and mucoadhesive properties of eye drops, which composition is based on hyaluronic acid. The in vitro test proved that hyaluronic acid with trehalose enhances the protection of human keratinocytes and increases the friction coefficient. The presented data can be used for the design of highly performing HA-formulations addressing specific needs before the preclinic.

The first review article “Hyaluronic Acid: A Key Ingredient in the Therapy of Inflammation” of our Special Issue summarises up-to-date articles (2017–2021) about the effect of hyaluronic acid and its influence on the inflammatory process [8], and it mostly aims to discuss the advances that have been achieved in the treatment of inflammatory diseases using hyaluronic acid as a key ingredient. The second review article is “Lung Hyaluronasome: Involvement of Low Molecular Weight Ha (Lmw-Ha) in Innate Immunity” [9], which is more focused on the specificity of low-molecular-weight hyaluronic acid in the lungs. The article summarises its role on pulmonary innate immunity and discusses the possible adverse effect and how to deal with them. The last review article “Endogenously Produced Hyaluronan and Its Potential to Regulate the Development of Peritoneal Adhesions” [10] deals with a specific but relatively common complication of intra-abdominal surgery. The authors are describing the role of hyaluronic acid in intra-abdominal healing, explaining on which base the peritoneal adhesions are developing and gives us the key to develop effective strategies against their formation.

Last article “Initial Observation of Factors Interfering with the Treatment of Alveolar Osteitis Using Hyaluronic Acid with Octenidine—A Series of Case Reports” [11] by Kapitán and his research group represent multiple case reports dealing with the treatment of alveolar osteitis which is quite common complication after the extraction of the third molars. These case reports series is based on the patient involved in a clinical trial described in “Hyaluronic Acid-Based Medical Device for Treatment of Alveolar Osteitis—Clinical Study” [12], which approved the pros and cons of using this device in the management of alveolar osteitis.

This Special Issue shows the wide potential usage of the Hyaluronic acid within the many different specialisation in human and non- human medicine. We believe that this insight can give hints to the reader such as how to potentially extend these applications, even in other medical fields, by similarities in the findings and enhancing the effectiveness of treatment.

## Data Availability

Not applicable.
